# Modulation of ODH Propane Selectivity by Zeolite Support Desilication: Vanadium Species Anchored to Al-Rich Shell as Crucial Active Sites

**DOI:** 10.3390/ijms23105584

**Published:** 2022-05-17

**Authors:** Małgorzata Smoliło-Utrata, Karolina A. Tarach, Katarzyna Samson, Mariusz Gackowski, Ewa Madej, Józef Korecki, Grzegorz Mordarski, Michał Śliwa, Sebastian Jarczewski, Jerzy Podobiński, Piotr Kuśtrowski, Jerzy Datka, Dorota Rutkowska-Zbik, Kinga Góra-Marek

**Affiliations:** 1 Jerzy Haber Institute of Catalysis and Surface Chemistry, Polish Academy of Sciences, Niezapominajek 8, 30-239 Krakow, Poland; malgorzata.smolilo-utrata@ikifp.edu.pl (M.S.-U.); katarzyna.samson@ikifp.edu.pl (K.S.); mariusz.gackowski@ikifp.edu.pl (M.G.); nczackie@cyf-kr.edu.pl (E.M.); jozef.korecki@ikifp.edu.pl (J.K.); grzegorz.mordarski@ikifp.edu.pl (G.M.); michal.sliwa@ikifp.edu.pl (M.Ś.); jerzy.podobinski@ikifp.edu.pl (J.P.); datka@chemia.uj.edu.pl (J.D.); 2 Faculty of Chemistry, Jagiellonian University in Krakow, Gronostajowa 2, 30-387 Krakow, Poland; karolina.tarach@uj.edu.pl (K.A.T.); jarczewski@chemia.uj.edu.pl (S.J.); piotr.kustrowski@uj.edu.pl (P.K.)

**Keywords:** vanadium, oxidative dehydrogenation, hierarchical zeolites, XAS, ^51^V MAS NMR, in situ IR, in situ UV-vis, 2D COS UV-vis

## Abstract

The commercially available zeolite HY and its desilicated analogue were subjected to a classical wet impregnation procedure with NH_4_VO_3_ to produce catalysts differentiated in acidic and redox properties. Various spectroscopic techniques (in situ probe molecules adsorption and time-resolved propane transformation FT-IR studies, XAS, ^51^V MAS NMR, and 2D COS UV-vis) were employed to study speciation, local coordination, and reducibility of the vanadium species introduced into the hierarchical faujasite zeolite. The acid-based redox properties of V centres were linked to catalytic activity in the oxidative dehydrogenation of propane. The modification of zeolite via caustic treatment is an effective method of adjusting its basicity—a parameter that plays an important role in the ODH process. The developed mesopore surface ensured the attachment of vanadium species to silanol groups and formation of isolated (SiO)_2_(HO)V=O and (SiO)_3_V=O sites or polymeric, highly dispersed forms located in the zeolite micropores. The higher basicity of HY_deSi_, due to the presence of the Al-rich shell, aided the activation of the C−H bond leading to a higher selectivity to propene. Its polymerisation and coke formation were inhibited by the lower acid strength of the protonic sites in desilicated zeolite. The Al-rich shell was also beneficial for anchoring V species and thus their reducibility. The operando UV-vis experiments revealed higher reactivity of the bridging oxygens V-O-V over the oxo-group V=O. The (SiO)_3_V=O species were found to be ineffective in propane oxidation when temperature does not exceed 400 °C.

## 1. Introduction

Propene is one of the most important substrates for many processes in chemical industry, in particular for the production of polymers. Currently, it is obtained mostly by steam cracking (SC), fluid catalytic cracking (FCC), or catalytic propane dehydrogenation (DH) using crude-oil-derived substrates [1]. The scarcity of natural resources on the one hand and the growing polymer market on the other will most likely lead to a significant increase in the demand for propene in the near future. However, the conventional propene production processes exhibit many disadvantages. Due to thermodynamic constraints, larger amounts of ethylene than propene are produced in the steam-cracking process. Industrial steam cracking is highly energy-consuming and produces CO_2_. Dehydrogenation suffers from a rapid deactivation of the catalyst by coke formation. Thus, finding competitive ways to produce propene has a high priority. One way to overcome the abovementioned problems is an oxidative dehydrogenation (ODH), in which alkane is converted into alkene in the presence of the oxidant, usually oxygen, but nitrous oxide or carbon dioxide can also be used [1,2,3]. The reaction is advantageous due to low energy consumption, lower coke formation, and higher selectivity to the desired product as compared with the conventional dehydrogenation process [4]. However, the process is still not in the commercialisation phase due to the difficulties in control of selectivity to olefins which results from overoxidation reactions that produce substantial amounts of undesired carbon oxides (CO_2_, CO). Indeed, the selectivity to alkenes decreases with the increase in alkane conversion, because the combustion rate of alkene is usually higher than the combustion rate of alkane [5]. To avoid overoxidation, it is necessary to develop highly structured materials where, on the one hand, the active phase is dispersed well enough to hinder subsequent alkene oxidation, but on the other hand its concentration allows for a fair ODH conversion rate.

Up to now, different types of catalysts have been tested in the oxidative dehydrogenation of alkanes. Most of them are based on transition metal oxides such as vanadium or molybdenum [6,7,8,9,10] or their mixed systems [11,12,13,14,15,16]. Recently, boron–nitride catalysts were also tested [17,18]. A lot of effort is put on vanadium-based systems as they are used in various types of oxidation reactions [19,20,21,22,23,24,25,26,27,28,29]. They are also employed in other areas such as gas sensors, electrochemical and optical switching devices, or reversible cathode materials for Li batteries [30].

The factors that govern the catalytic performance of the vanadium-based systems are, however, still being debated. Advanced studies performed on vanadium-containing materials have shown that the rate of propene formation is correlated with the dispersion of the VO_x_ species and a local coordination of the vanadium ions [9,19]. The presence of highly dispersed vanadium species in which the V ions experience tetrahedral or pseudo-tetrahedral coordination usually enhances selective oxidation reactions, whereas the presence of polynuclear pseudo-octahedral vanadium species is attributed to higher conversions and promotes combustion [21,31]. The acidity of the support on which vanadium phase is deposited is also important. Usually, acid sites of moderate strength are beneficial for the ODH activity, whereas too strong ones enhance aromatization [32]. Last but not least, some claim that the pivotal role is played by the properties of oxygen atoms which are directly connected to vanadium atoms [19]. Their different forms, often considered as electrophilic or nucleophilic reaction centres, are usually responsible for total oxidation or selective oxidation, respectively.

Vanadium-loaded zeolites and other mesoporous materials proved to be competitive systems in the ODH of light alkanes in view of the abovementioned issues relating vanadium activity with geometry properties. The catalysts composed of micro- and mesoporous materials as supports for the vanadium active phase shows a great advantage due to the high activity and remarkable stability [33,34,35]. The existence of pores of relatively small diameters facilitates high dispersion of vanadium and impedes the growth of long-chain polymeric vanadium clusters. Finally, zeolites exhibit acid sites whose presence is of importance for the selectivity in the ODH process. One often invokes the problem of diffusional limits as the main disadvantage of the zeolitic supports; however, this can be overcome by the use of hierarchical or hierarchized zeolites, in which mesopores are introduced, leading to improved mass transport to and from the active sites located in the micropores [36].

In the present study, we decided to offer a deep insight into the state of vanadium and its interaction with the surface groups of the zeolitic supports as well as to follow the acidity changes upon desilication of the catalysts and to link these parameters with the observed catalytic performance. The X-ray absorption spectroscopy (XAS), nuclear magnetic resonance (^51^V MAS NMR), and two-dimensional correlation ultraviolet—visible spectroscopy (2D COS UV-vis) techniques to probe the oxidation state and the local coordination of vanadium ions were employed. The temperature-programmed reduction with hydrogen (H_2_-TPR) was applied to study the reducibility of the active phase, and in situ Fourier Transform Infrared Spectroscopy (FT-IR spectroscopy) with probes/reagent molecule was used to assess the acid-based property of the V-catalysts.

## 2. Results and Discussion

### 2.1. Structure, Morphology, and Textural Properties

**Figure 1:**
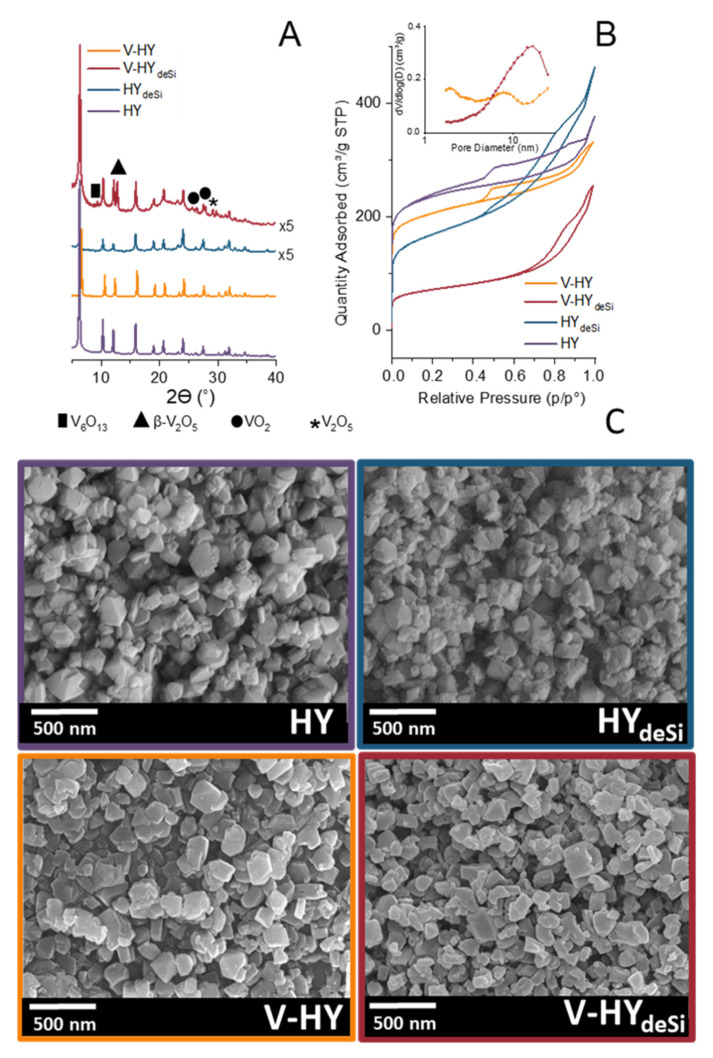
The XRD patterns (**A**), N_2_ physisorption isotherms with pore size distribution (inset) (**B**), and SEM pictures of V- and H-zeolites (**C**).

The protonic forms of the studied zeolites are denoted as HY and HY_deSi_ while their vanadium-loaded counterparts are denoted as V-HY and V-HY_deSi_. The X-ray diffraction (XRD) analysis confirmed the crystallinity of the parent HY zeolite and its desilicated counterpart (Figure 1A). The HY_deSi_ support exhibits lower crystallinity compared with HY but, what should be underlined is that no amorphous phase was observed upon desilication due to an effective transport of the extracted phase through the mesopore system generated by steaming and the subsequent dealumination process during the pristine material preparation. In the desilicated material the share of smaller crystallites increases what is anticipated from broadening of the diffraction peaks for HY_deSi_. Preserving of the crystallinity upon extensive desilication clearly points to the utility of the zeolite in catalytic or adsorption applications. The extent of the desilication process is well documented by the decreased Si/Al ratio (18 for HY_deSi_ vs. 31 for HY–Table 1). The enrichment in aluminium species, i.e., a presence of re-aluminated Al species [37,38] on the surface of HY_deSi_ can have an impact not only on the structural parameters of the zeolitic support but also on the formation of vanadium centres, different from those for HY deprived of the Al-rich shell.

The zeolitic crystalline structure of the pristine HY material is preserved upon dispersion of the vanadium phase. The absence of XRD peaks from vanadium species point to their amorphous nature or formation of the crystallites of the vanadium phase small enough to be below the XRD detection limit. In contrast, the wet impregnation procedure of the vanadium species deposition on the desilicated zeolite results in an appearance of the silica–amorphous phase to some extent. This effect is not surprising as the re-aluminated surface of HY_deSi_ is susceptible to the acidic environment (applied in the vanadium phase deposition stage). The presence of vanadium crystalline moieties is identified in V-HY_deSi_ by the additional peaks at 2θ = 9°, 12°, 25°, 27°, 29° attributed to V_6_O_13_, β-V_2_O_5_, VO_2_, and V_2_O_5_, respectively (Figure 1A) [39].

All the studied catalysts are micro–mesoporous materials, presenting isotherms of type IV (Figure 1B). Obviously, the caustic treatment resulted in the upsurge in the mesoporous characteristics: the mesopore volume increased from 0.22 cm^3^/g (for HY) to 0.53 cm^3^/g (for HY_deSi_) and the specific surface area related to mesoporosity, S_meso_, raised from 158 m^3^/g (for HY) to 423 m^3^/g (for HY_deSi_). The incorporation of the vanadium species influenced at most both micro- and mesoporous characteristics of V-HY_deSi_. The lowering of V_micro_ and S_meso_ by 60% of H-zeolites values was attributed to the preferential location of the V species in micropores and partial amorphization of this material upon wet impregnation procedure. The latter finding is in line with the XRD data. The pore size distributions determined by BJH model (Figure 1B, inset) documents the fact that the V-loaded desilicated zeolite V-HY_deSi_ contains the secondary mesoporosity with a relatively narrow mesopore diameter distribution centered at ca. 11 nm.

The SEM micrographs showed that the desilication process seems to increase the share of smaller grains in HY_deSi_ and V-HY_deSi_, which can be related with the alkaline treatment primary targeting the intergrowth boundaries rich in defects (Figure 1C). Moreover, the smooth grain surfaces and not distinguishable amorphous phase in V-HY_deSi_ justifies the inference of the amorphous phase persistence inside the grains.

### 2.2. Acidity FT-IR Characterization with CO and Pyridine as the Probes

**Figure 2:**
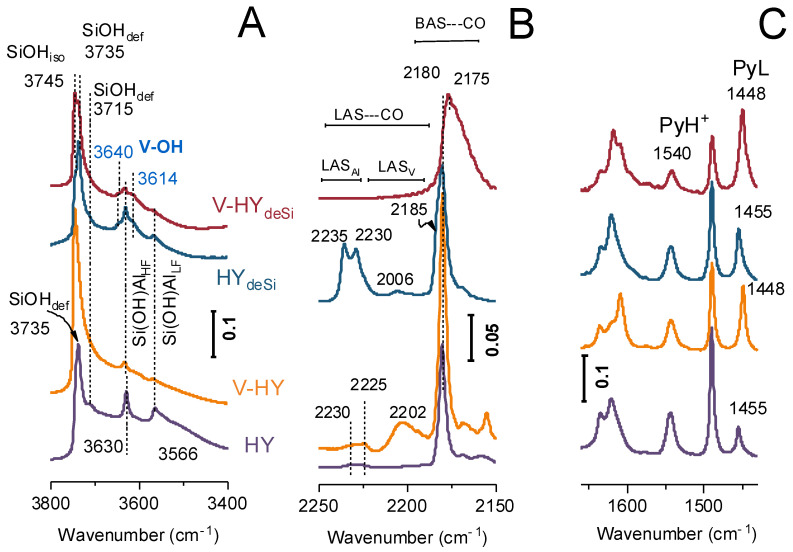
The FT-IR spectra of the V zeolites and their H counterparts: in the region of O-H stretching vibrations (**A**), upon the sorption of carbon monoxide (**B**) and pyridine (**C**).

Alterations in the nature of the surface OH groups (Figure 2A) caused by both the desilication process and the deposition of the vanadium phase constitute the first clue concerning the location and the attachment of the vanadium sites to the zeolite surface.

The silanols are highly populated on both the external surface (Si-OH, 3745 and 3735 cm^−1^) and in the micropore defects or intergrowth boundaries (3715 cm^−1^) of the pristine HY zeolite. Among silanol species located on external surface, those perturbed by hydrogen bonding (Si-OH_def_, 3735 cm^−1^) are represented in majority. The highly acidic Si(OH)Al sites oscillating in supercages and in the hexagonal prisms are recognized by 3630 cm^−1^ (Si-OH-Al_HF_) and 3550 cm^−1^ (Si-OH-Al_LF_) bands, respectively. Upon caustic treatment the abundance of the isolated external Si-OH groups significantly increased what is manifested as the appearance of the well-resolved band at 3745 cm^−1^, and the important development of S_meso_ (Table 1). The intensity of the Si(OH)Al groups’ bands reduction upon desilication is unambiguously related with the lowered number of tetrahedral Al atoms able to generate the Brønsted sites. The process of HY_deSi_ zeolite surface realumination is very advanced, which is documented by a decrease in Si/Al (from 31 to 18). Nevertheless, Al atoms reinserted onto mesopore walls are very unstable in tetrahedral positions and susceptible for dehydroxylation [38]; thus, the conversion in Lewis sites during thermal treatment occurred. Indeed, in HY_deSi_ the presence of important number of electron acceptor sites of very high acid strength, identified by the 2235 and 2230 cm^−1^ bands, is confirmed by the results of the low-temperature sorption of carbon monoxide (Figure 2B).

The IR signatures for V-HY and V-HY_deSi_ indicate the interaction of the isolated silanols with the vanadium species: the 3745 cm^−1^ band diminishes upon deposition of vanadium. This effect is peculiarly pronounced for vanadium dispersed on the desilicated zeolite. The silanols experiencing polarizing effect from the dispersed vanadium species can be identified by the 3735 cm^−1^ band. The strong Si-O^δ−^⋯^δ+^V interactions lead to the appearance of hydroxyls of the Brønsted acidic character identified by the band at 3614 cm^−1^ [40]. In addition, another type of a V-originated Brønsted acid site is clearly visible in the V zeolites. The 3640 cm^−1^ band, assigned to the V-OH [40,41] vibrations, was previously observed for vanadium supported on TiO_2_ [42] and on titania/silica [43] materials. The newly created sites can be considered as the (SiO)_3_V=O or (SiO)_2_(HO)V=O species, as will be evidenced in the ^51^V MAS NMR experiments (Section 2.3). The replacement of protons in Si(O^−^)Al with positively charged vanadium oxo species resulted also in elimination of some Brønsted acid sites, as manifested by lowering of the intensity of the Si(OH)Al bands by ca. 50% in both V zeolites. Indeed, the CO sorption FT-IR data confirm the formation of new electron acceptor sites (CO band at 2206 cm^−1^) upon V-deposition in the HY zeolite. For V-HY_deSi_ the lack of an effective ligation of weak basic CO to vanadium phase is found. Such behaviour is most likely due to the low electron acceptor properties of the vanadium sites formed on the surface of the desilicated zeolite. Formation of new phases of the Lewis acidic character of a very low strength (not detectable with CO) can be assigned to the interaction of Al cations from re-aluminated surface of HY_deSi_ with the supported vanadium oxo species. Such V-Al-O systems were found to possess essentially redox activity [44].

The overall acidic property picture was obtained from quantitative Py sorption FT-IR studies. Starting from Al molar concentration of 430 μmol·g^−1^ for the HY, the 303 μmol·g^−1^, i.e., 70% of the Al atoms are detected by Py sorption (Table 1, B + L, sum of Brønsted and Lewis sites). The caustic leaching reduced the number of protonic sites by 18% producing a twofold higher number of the Lewis sites. The deposition of the V species contributed to the consumption of the Brønsted acid sites by ca. 70% and 45% in V-HY and V-HY_deSi_, respectively. At the same time, the number of Lewis sites, which can be assigned to the vanadium centres, increased. Assuming that the deposition of the V-phase implies the appearance of additional Lewis centres, the concentration of the Lewis V sites was calculated as the difference between the number of Lewis centres in the V and H zeolites (Table 1, LAS_V_). In terms of absolute values, the V-HY possess the highest concentration of V-originating electron acceptor sites. One should have in mind, however, that the concentration of the native Lewis sites in the HY_deSi_ zeolite (those originating from the aluminium atoms) was affected by the formation of new Lewis acidic property of the V-Al-O-phases of a very low strength [44]. However, the share of vanadium sites able to bond Py expressed per 1 m^2^ is still higher for V-HY_deSi_. The abovementioned considerations are fully consistent with the results of the CO sorption. Furthermore, the inspection of the spectra of Py interaction with the surface acid sites clearly points to the appearance of the weak acid sites in both V-HY and V-HY_deSi_. The strong Lewis acid sites (PyL adduct band at 1455 cm^−1^) in H-zeolites are replaced with the V sites of significantly lower strength (PyL adduct band at 1448 cm^−1^) (Figure 1C). The significant drop of the strength of the protonic sites both in the desilicated and the vanadium-deposited materials is manifested by the Py thermodesorption data (BAS Py_450_/Py_170_^b^–Table 1).

### 2.3. On the Nature of Vanadium Species

**Figure 3:**
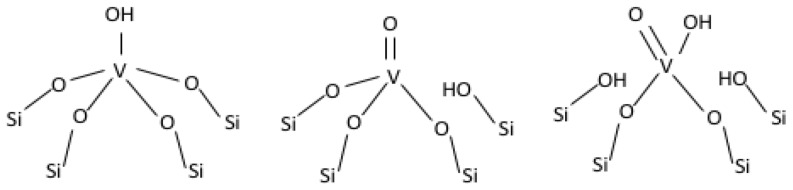
Different forms of vanadium species.

The central T atom in the zeolite matrix can be replaced with vanadium species in such a way that its coordination sphere is saturated with the Si tetrahedra. Three different forms of vanadium species are therefore considered: V–OH, V=O, and V(=O)(–OH)—see Figure 3. In all of the models, vanadium was on +5 oxidation state and formed, respectively, 4, 3, and 2 bonds with the neighbouring Si atoms via oxygen bridges. The V–OH and V=O centres were proposed based on the available literature data on the geometry and environment of the V sites in zeolites [45,46,47,48].

**Figure 4:**
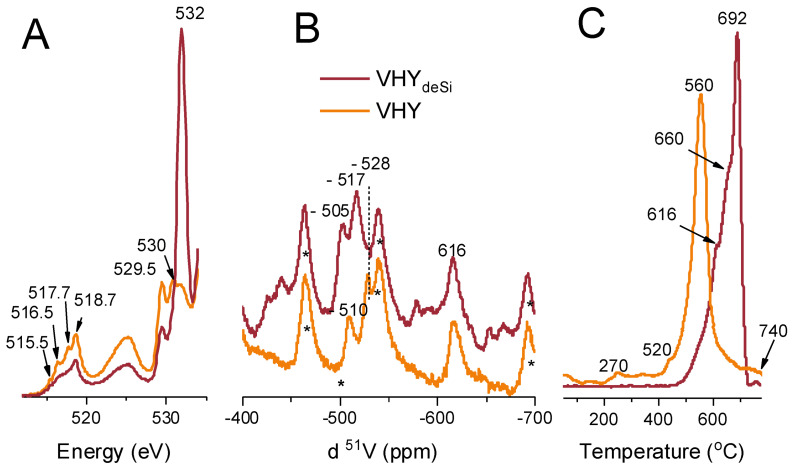
Partial fluorescence yield XAS spectra of V L_3,2_ and O K edge regions for vanadium supported on zeolites studied (**A**). ^51^V MAS NMR spectra (**B**). *—denotes spinning side bands. H_2_-TPR profiles (**C**).

Figure 4A presents XAS spectra for vanadium supported on the pristine HY (A) and the desilicated HY_deSi_ (B) zeolites. The measured XAS spectra are split into three-line groups. The vanadium lines reflect the spin–orbit splitting (approximately 6.7 eV) and comprise the V L_3_-edge in the energy range 515–518 eV, corresponding to transitions from V 2p_3/2_ to V 3d, and the V L_2_-edge, at approximately 525 eV, corresponding to transitions from V 2p_1/2_ to V 3d. The third group, above 528 eV, is the O-K-edge, corresponding to transitions from O 1s to O 2p.

For the V-HY_deSi_ spectra, the V L_3_-edge shows well resolved lines at 515.5, 516.5, 517.7, and 518.7 eV. Such a rich structure is reported for V_2_O_5_ [49,50] and attributed to crystal field splitting of unoccupied V 3D orbitals. For disordered vanadium oxides, such as powder and polycrystalline samples, the structure of the L_3_ edge is less distinct [51], which is the case of desilicated V-HY_deSi_ zeolite (Figure 1B). The O-K-edge spectrum region below 535 eV is determined by O-V bonding [52]. The lines at 529.5 eV and 532 eV correspond to the electronic transitions from the O 1s core to the O 2p–V 3d(t_2g_) and O 2p–V 3d(e_g_) states, respectively [53]. A striking feature for the V-HY_deSi_ zeolite is the domination of the 532 eV line, the effect being much weaker for V-HY. This finding indicates for the changes of the chemical vanadium state when it is dispersed on the zeolitic supports of various acidic characteristics are most probably related to the presence of the V^4+^ species. An alternative explanation had to involve a strong hybridization to the e_g_ substates in V-HY_deSi_ as the lines at 529.5 eV and 532 eV corresponds to the hybridization between O2p and V3d t_2g_ and e_g_, respectively. 

A solid-state ^51^V MAS NMR is a suitable technique to investigate supported V^v^ oxides since the nuclear spin of vanadium is 7/2 and its natural abundance is almost 100%. Consequently, we used ^51^V MAS NMR capability to discriminate between different coordination environments of vanadium oxides (Figure 4B). The literature reports two narrow peaks emerging at ca. −510 ppm and −528 ppm, arising from the extra-framework vanadium species in less- and more-distorted pseudo-octahedral coordination, respectively [54,55,56], as was postulated for vanadium-substituted BEA zeolite or vanadium-loaded MCM-41 mesoporous material [54,57]. The signal at −616 ppm (bigger shielding effect) can be indicative for the presence of V^5+^ in the pseudo-tetrahedral positions (isolated or polymerised tetrahedra), i.e., (SiO)_3_V=O or (SiO)_2_V=O(OH) [57,58]. The shift of the distorted octahedral V species line position in V-HY_deSi_—in respect to the ^51^V resonance line of the V-HY material—can be also considered as a sensitive reporter of the electronic state of either the vanadium(V) site or its neighbourhood. In the V-HY_deSi_ material the −528 ppm signal of the less deshielded pseudo-octahedral V sites is absent while a new signal of −517 ppm arises in the V-HY_deSi_ material. The downfield shift indicates for lower shielding effect resulting from a decreased electron density around a V nucleus ruled both by the altered Si/Al ratio of zeolitic framework and by the realumination process during the desilication. Indeed, the oxygen atoms in a zeolite lattice have an intrinsic Lewis base character due to the electron pairs they can donate and to the partial negative charges they bear. The strength of such a donation is partially reduced by Si and Al atoms which provide partial positive charges. Since Al and Si atoms differ in the electronegativity, the increased number of Al atoms in the zeolitic framework induces higher basicity of the framework oxygen atoms. Thus, the higher basicity of the HY_deSi_, in comparison with its non-desilicated counterpart HY support, is explained in light of the primary dependence of the negative charge borne by oxygen framework atoms on the chemical composition of the zeolite, i.e., the Si/Al ratio [59]. Furthermore, the higher partial charge of the framework oxygen is responsible for a removal of electron density from the V atoms resulting in a more pronounced deshielding of the latter in V-HY_deSi._ This effect is even enhanced for V species interaction with the Al-rich shell.

The values V^(oct)^/V^(tetra)^ ratio extracted from the NMR spectra (0.95 for V-HY and 1.30 for V-HY_deSi_) clearly documents that a relative share of pseudo-octahedral V species is higher in the V-HY_deSi_ sample, which corresponds well with the lower intensity of FT-IR bands at 3640 and 3613 cm^−1^ for V-OH groups in this material. In the V-HY_deSi_, the main contribution of the pseudo-octahedral V species is related to more organized surface structures, detected by XRD.

An insight into the reducibility of the surface vanadium species was derived from the temperature-programmed reduction measurements (Figure 4C) since H_2_-TPR profiles strongly depend on a vanadium oxidation state, the VO_x_ surface loading, and a strength of the interaction of the V-containing species with a support [60,61]. Centi et al. reported for V-silicalite that the temperature of the maximum rate of reduction was related to the strength of the V-O-M bond (e.g., M = Si, Ti, Al) more than to the coordination of vanadium [42]. The extra-framework oligomeric species (maximum rate of reduction at 440 °C) or the dispersed vanadium species located in the zeolite channels (maximum rate of reduction at 480–560 °C) appeared to be less resistant to reduction with H_2_ than the framework V^5+^ species, which reduced to V^4+^ gave a peak at ca 570–680 °C. The deep reduction in framework V^5+^ to V^3+^ required much higher temperature and occurred above 725 °C [62,63]. In the H_2_-TPR profile of the studied V zeolites, the reduction peaks spread in the temperature range of 270–740 °C. For V-HY, the main reduction peak was centred at 560 °C, at temperature significantly lower than that observed for V-HY_deSi_. Its lower complexity points to the similar reducibility of surface vanadium species in V-HY, i.e., the framework V^5+^ species accompanied by dispersed extra-framework ones, but the latter are not located in the micropores. Such reducibility characteristics of the V-HY material is supported by the N_2_-sorption data (see Figure 1B) evidencing only a small decrease in microporous and mesoporous features of V-HY upon the V deposition. The reduction in vanadium species dispersed on the desilicated support, V-HY_deSi_, is realized via several steps and at significantly higher temperatures (the main peak at 692 °C), which justifies the assumption on the strong interaction of vanadium V^5+^ with the zeolite framework. Due to the presence of XRD peaks, characteristic of crystalline vanadium phases (Figure 1A), their contribution to the H_2_-reduction profile at 692 °C cannot be discarded, which further explains a complexity of the reduction peak. For the vanadium silicalite–1 (VS–1) a possible product for reduction of V^5+^ in the framework was V^4+^, as identified by the reduction peak at 570–680 °C [63]. Therefore, the appearance of the peak at 692 °C was ascribed to the reduction V^5+^ anchored to the surface-Al-rich shell, i.e., the (SiO)_3-n_(AlO)_n_V=O. The 616 and 660 °C peaks are believed to originate from the V^5+^ to V^4+^ reduction of the species incorporated to siliceous fragments of zeolite framework (the (SiO)_3_V=O) as the interaction of vanadium oxo species with alumina was found to be stronger than with silica, which resulted in lower mobility of surface vanadium oxide species on the former carrier. The engagement of the silanols groups in the attachment of vanadium species, especially to HY_deSi_ surface, was previously evidenced in IR studies. The results provide the evidence on high dispersed monomeric VO_4_ and oligomeric VO_x_ species in both catalysts, highlighting that the supports had different effects on the dispersion of the oxides and thus ultimately determine the type of oxygen species associated with the vanadium centres, i.e., the bridging oxygen sites (V-O-V, V-O-Al) and the terminal V-OH bonds.

**Figure 5:**
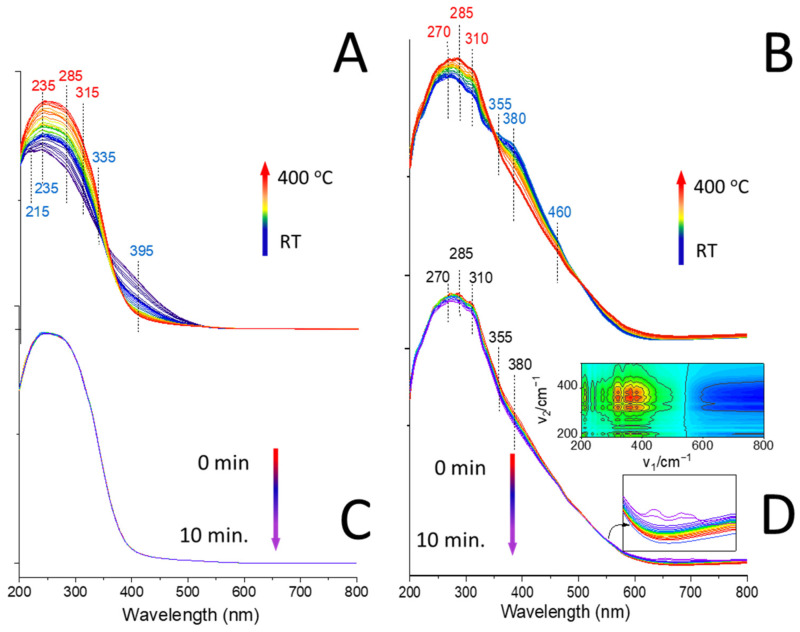
The DR UV-vis spectra of V-HY (**A**,**C**) and V-HY_deSi_ (**B**,**D**) collected during the thermal pretreatment in synthetic air flow (**A**,**B**) upon contact with propane at 400 °C (**C**,**D**). The 2D COS UV-vis maps as inserts.

The diffuse-reflectance UV-vis spectroscopy (DR UV-vis) provided additional insight into the different oxidation states and coordination geometries of the vanadium species dispersed on the studied zeolites. Using operando UV-vis, the charge transfer transitions as well as the d–d transitions of vanadium ions at the catalyst surface were probed upon the contact with air and propane at elevated temperatures. Figure 5 displays the spectra of the V-catalysts collected upon thermal treatment, during which the temperature was stepwise increased at a rate of 6 °C/min and kept for 10 min at each temperature. In nonthermally pretreated V-HY_deSi_, two bands at 260 and 320 nm originating from monomeric tetrahedral V^5+^ species [64] and attributed to π(t2) → d(e) and to π(t1) → d(e) oxygen–tetrahedral V^5+^ charge transfer transitions due to the presence of pseudo-tetrahedral O_3/2_V=O species, anchored to the zeolitic walls and possessing a V=O double bond, i.e., the (SiO)_3_V=O or (SiO)_2_(HO)V=O species, can be distinguished. The band at 380 nm has been attributed to dispersed polymeric vanadium(V) sites either tetrahedral or pentahedral coordination [64]. The agglomerates of the pentacoordinate V_2_O_5_ (∼460–500 nm) are poorly populated. The pretreatment at air atmosphere at increasing temperature induces a strong shift of the UV-visible bands to lower wavelength. The observed effects are associated with dehydration of the catalyst surface and can be ascribed to modification of symmetry: the V-HY_deSi_ fully dehydrated at 400 °C showed the intense band at 270 nm (with a shoulder at 315 nm) of tetrahedral V^5+^ formed at the expense of 375 cm^−1^ band (octahedral vanadium species). Thermal pretreatment of V-HY induces stronger than for V-HY_deSi_ shift of the UV-visible bands to lower wavelengths, i.e., from 400 nm to 215–310 nm. Differentiated intensities of newly created bands point to a greater susceptibility of V species in V-HY for water elimination. 

After contact with propane at 400 °C, the solely V-HY_deSi_ catalyst was partially reduced what is manifested by the decrease in the 270–380 nm band of the tetrahedral and octahedral V^5+^ species and accompanied by the evolution of the broad band centred at ca. 700 nm. These d–d charge transfer absorption bands are typical of VO_2_ (V^4+^): 625 nm for the b_2_(d_xy_) → b_1_(d_x2-y2_) transition and 770 nm for the b_2_(d_xy_) → e(d_xy_,d_yz_) transition [65]. It is well known that the origin of the specific electronic transition for vanadium ions is sometimes difficult to isolate due to its dependence on the local coordination environment, the polymerization degree, and the specific oxidation state, particularly when the observed effects are not reflected in the intensity. Therefore, the formation of V^4+^ at the expense of V^5+^ was proven using 2D COS analysis (Figure 5D, inset). The use of 2D COS analysis significantly enhances the use of IR and UV-vis spectroscopies [66,67,68], allowing for interpretation of even the slightest changes in the spectra during heterogeneous reactions, even at high temperatures. Indeed, the 2D COS UV-vis maps allow for differentiating the subtle differences in the time-dependent intensity variations of the UV-vis bands which are poorly noticeable in 1D spectra. The positive correlation between two separate wavenumbers presents the simultaneous increase or decrease in intensity of these bands, while the negative correlation documents the changes of the band’s intensity at the expense of the other one. Despite the apparent decrease in the intensities of the 270 and 285 nm bands, this effect is not correlated with the V^4+^ formation. The sites represented by bands at 320, 360, and 375 nm are the only species that are consumed in the presence of propane and accompanied by the evolution of the V^4+^ bands, as manifested by their negative correlations. According to the literature, higher reactivity of the bridging oxygens V-O-V than the oxo-group V=O [69] was agreed at lower temperatures. Based on the operando UV-vis results, the 320 and 360 nm were ascribed to the (SiO)_2_(HO)V=O and the highly dispersed polymeric tetrahedral vanadium(V) sites, respectively, while the 375 nm band was ascribed to the octahedral species, also active in the ODH process. The (SiO)_3_V=O species identified by 270–285 nm bands were found to be ineffective in propane oxidation when temperature does not exceed 400 °C. Indeed, the V-HY, in which these species are the most abundant, provides marginal catalytic activity, as shown in Section 3.6. The involvement of propane activation on the latticed pseudo-tetrahedral vanadium species according to the Mars van Krevelen mechanism was unquestionably proven as shown by theoretical and experimental studies [10,35,70,71]. Therefore, the possibility of using the 2D COS analysis of UV-vis results for identifying the vanadium centres in zeolites responsible for propane transformation, to the best of our knowledge, was demonstrated for the first time.

### 2.4. In Situ FT-IR Studies of ODH Process

**Figure 6:**
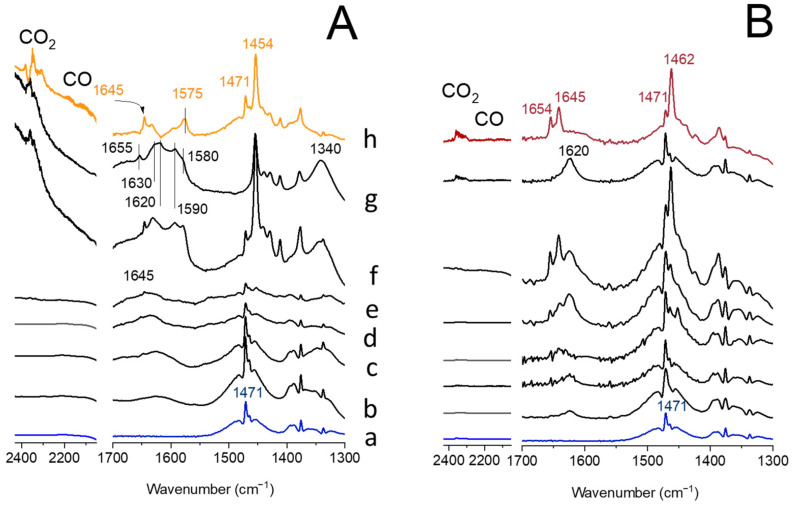
In situ FT-IR spectra collected during propane ODH process over V-HY (**A**) and V-HY_deSi_ (**B**) presented in 2400–2050 cm^−1^ (CO and CO_2_) and 1700–1300 cm^−1^ (surface adsorbed species) spectral ranges: gaseous propane at RT (a), propane on the catalyst at RT (b), propane and O_2_ (c) at RT, (d) at 200 °C, (e) at 350 °C, (f) upon reaction at 350 °C and cooled down to RT, (g) upon desorption at RT, and (h) the spectrum of gaseous species formed during process.

The spectroscopic studies were supplemented by in situ FT-IR measurements of propane and O_2_ (stoichiometric ratio) transformation in the presence of the V-catalysts. In situ FT-IR spectra obtained during propane ODH process at increasing temperature are presented in 2400–2250 cm^−1^ and 1700–1280 cm^−1^ ranges to show the formation of CO/CO_2_ and surface adsorbed species, respectively (Figure 6). Upon introduction of propane at room temperature over the V-HY, only its gaseous phase (1471 cm^−1^) was detected (Figure 6A, spectra a and b). No detectable changes were identified when the O_2_ co-reagent was introduced at RT: gaseous propane still dominates (spectrum c). The propane oxidation starts at 200 °C (spectrum d), as manifested by the weak oscillation–rotational spectrum of CO (2300–2100 cm^−1^), and CO_2_ (2345 cm^−1^). Among the adsorbed species, the formate and acetate (1590–1570 cm^−1^), acrylate (1655 cm^−1^), carbonates (1340 cm^−1^), and water (1620 cm^−1^) were recognized in the FT-IR spectrum collected at RT upon ODH process preformed for 10 min at 350 °C (spectrum f). The activity of V-HY toward stabilization of the reaction products (spectrum g) was already apparent, while propene (1645 cm^−1^), CO, and CO_2_ were identified in gaseous products (spectrum h). The stability of alkoxide species is significantly lower in V-HY_deSi_, because, in the presence of vanadium sites anchored to the Al-enriched (realumination) surface of the support, they are immediately transformed to propene, water, and CO/CO_2_, since only these species accompanied by the unreacted propane are identified in the FT-IR spectra (Figure 6B, spectrum d).

### 2.5. Catalytic Properties of the System

**Figure 7:**
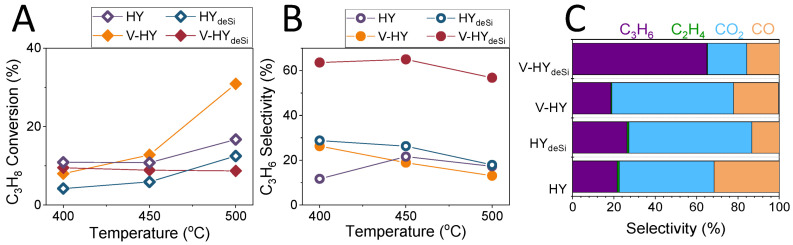
Conversion of propane (**A**), selectivity to propene (**B**) and propane ODH products distribution determined for the H- and V-catalysts at various temperatures (contact time 0.33 s) (**C**) [35].

The V zeolites were tested in the oxidative dehydrogenation of propane and their catalytic activity was then discussed in terms of the speciation of vanadium sites. Figure 7A illustrates a performance of the V catalysts in different reaction temperatures. Attained low conversions (below 40%) in the studied temperature range (400–500 °C) are typical for the studied process [61,72]. The V catalysts show comparable conversion rates: 8.9% and 12.8% for V-HY_deSi_ and V-HY, respectively. Similar conversion at 400 and 450 °C is also observed for the native HY evidencing that the ODH active V sites are not offered by the V-HY material at these temperatures.

Figure 7B shows a distribution of the reaction products determined for the studied catalysts at 450 °C at the contact time of 0.33 s. The maximum selectivity of 65.0% was achieved for propene using V-HY_deSi_ at 450 °C. On the other hand, 18.9% selectivity for propene was observed at 450 °C when the same amount of vanadium was deposited on V-HY. These are mostly carbon oxides (CO and CO_2_) which are formed because of propane total oxidation. Ethylene resulting from the propane cracking is found at considerably smaller amounts. The higher selectivity towards propene is observed for V-HY_deSi_, while the lowest one is found for V-HY. The latter did not offer better selectivity than the pristine HY evidencing that only the peculiar V sites providing selectivity to propene via ODH are present in the V-HY_deSi_ material.

The comparison of the measured propene selectivity with the literature data is possible for similar systems. For instance, the V-HY_deSi_ is competitive with V-MFI and V_6_ITQ-6 composites as they reach 52% and 60% C_3_H_6_ selectivity at considerably higher temperature (550 °C) while with the same conversion (ca. 10%) [35,73,74].

According to the Mars van Krevelen mechanism, the ODH process starts from a weak associative adsorption of propane on lattice oxygen which is followed by a C−H cleavage via H-abstraction from propane using a neighbouring lattice oxygen. Hydride elimination from adsorbed alkoxide species produces propene which desorbs while OH groups undergo recombination to form water and the reduced M centre. The cleavage of C−H bonds in alkanes is ruled by the basicity of the lattice oxygen anions that abstract the H atoms [75]. The higher activity and selectivity of V-HY_deSi_ can be explained by higher basicity of HY_deSi_ than HY support dictated by lower the Si/Al ratio of the former one, but, first and foremost, by the presence of the Al-rich shell (realumination) which helps the C−H bond activation. As was shown in FT-IR studies of propane and O_2_ transformation (Figure 6) the stability of oxo- and propane-derived species is also lower in V-HY_deSi_ than in V-HY. As a result, a rate of desorption of propene is significant. In contrast, high accumulation of acetate and formate species on the V-HY surface (Figure 6) points to the retaining adsorbed propane intermediates on the surface, thus their undesired oxidation to the CO_x_ products, in line with the selectivity data. When considering the reaction path, the propene can be adsorbed again on the surface and get oxidized to isopropoxide species [76,77,78] through a neighbouring V-OH as the Brønsted acid site and continues with the subsequent oxidation steps. In V-HY_deSi_, the lower concentration of the V-OH species hampers the undesired oxidation reactions while lower strength of the protonic sites inhibits polymerization of propene and coke species formation. In the final step of the ODH reaction, the reduced metal site is reoxidized via dissociative chemisorption of O_2_ [76,79]. The reducibility of the catalyst therefore plays the decisive role in the ODH process. When the reduction steps become more facile than reoxidation, the surface of catalysts becomes rapidly oxygen-poor and, as a result, easily reducible oxides can be ineffective in the ODH reactions. This is the case of the V-HY zeolite.

## 3. Materials and Methods

### 3.1. Synthesis

Vanadium catalysts with 6 wt.% nominal content were prepared by wet impregnation at pH = 2.5 using water solution of ammonium metavanadate (NH_4_VO_3,_ POCh, ACS reagent grade) as the source of vanadium. The protonic form of super-dealuminated, ultra-stabilized zeolite (HY) supplied by the Zeolyst International Company, Farmsum, The Netherlands (CBV 760, Si/Al = 31), and the zeolite desilicated by 10% mixture of tetrabutylammonium hydroxide (t-BAOH, POCh, Avantor Performance Materials Poland S.A., Gliwice, Poland, ACS reagent grade) and 0.2 M NaOH (POCh, Avantor Performance Materials Poland S.A., Gliwice, Poland, ACS reagent grade), yielding the HY_deSi_ of Si/Al ratio of 18, were used as supports. The desilicated HY_deSi_ before the vanadium deposition was subject to ion-exchange procedure to obtain ammonium form. The zeolite sample was immersed into 1 M solution of NH_4_NO_3_ for 2 h at 80 °C and centrifuged. The ion-exchange procedure was repeated five times. The resulting support was dried for 12 h at room temperature and calcined in air flow for 10 h at 500 °C. The Si/Al ratios were determined by the XRF measurements. The resulting samples, further denoted as V-HY and V-HY_deSi_, respectively, were calcined in an air flow for 8 h at 500 °C.

### 3.2. X-ray Diffraction (XRD)

The structure of the prepared materials was determined by a Rigaku Multiflex diffractometer (Rigaku, Tokyo, Japan) equipped with Cu Kα radiation (40 kV, 40 mA). The measurements were performed in the 2θ range from 5 to 50° for the zeolitic catalysts, with a scan speed of 1 deg min.

### 3.3. X-ray Fluorescence (XRF)

The XRF spectroscopy measurements were performed using EDX 3600H apparatus by Skyray Instrument Inc., Stoughton, MA, USA, equipped with tungsten lamp of 9 kV and 40 kV voltage to determine Si and Al as well as V, respectively.

### 3.4. Scanning Electron Microscopy (SEM)

High magnification SEM images were recorded using a JEOL JSM-7500F Field Emission Scanning Electron Microscope (SEM) (JEOL Ltd., Tokyo, Japan) equipped with the X-ray energy dispersive (EDS) system—INCA PentaFetx3 (Oxford Instruments Analytical, High Wycombe, UK). The secondary electron detector provides SEI images, and the back scattered electron detector provides BSE (COMPO) micrographs. K575X Turbo Sputter Coater was used for coating the specimens with chromium (deposited film thickness—20 nm).

### 3.5. X-ray Absorption Spectroscopy (XAS)

To probe the vanadium oxidation state, synchrotron X-ray absorption experiments were done. Measurements of X-ray absorption spectra (XAS) at the V L_2,3_ and O K edges were performed at the National Synchrotron Radiation Centre SOLARIS in Krakow, at the bending magnet XAS/PEEM beamline [80]. The spectra were collected at the XAS end-station in the partial fluorescent yield (PFY) detection mode using a silicon drift detector. The X-ray energy was calibrated with an accuracy of ±0.1 eV. XAS applied to systems containing low amounts of VO_x_ groups allows to provide important information of the vanadium oxidation state keeping signals from the V L_2,3_ and O K edges separated and easier to interpret, in contrast to X-ray photoelectron spectroscopy (XPS). Recently, the technique gains popularity in heterogeneous catalysis [81].

### 3.6. Nuclear Magnetic Resonance (^51^V MAS NMR)

Local coordination of vanadium ions was probed by ^51^V MAS NMR spectra recorded using Bruker Avance III 500.13 MHz (11.7 T) spectrometer (Bruker, Billerica, MA, USA). The samples were spun in 4 mm zirconia rotor with variable speeds from 5.5 to 10 kHz. The spectra were acquired with single-pulse excitation using 0.6 µsec pulse (π/8) operating at 131.57 MHz of resonance frequency. In a typical run, 122,880 scans were recorded. Chemical shifts are referenced to VOClO_3_ using secondary references NH_4_VO_3_ (d = 570 ppm).

### 3.7. Temperature Programmed Reduction with Hydrogen (H_2_-TPR)

H_2_-TPR measurements were carried out on a Quantachrome Chembet 3000 apparatus (Anton Paar GmbH, Graz, Austria) in the temperature range 25–800 °C. A measure of 100 mg of a sample placed in a U-shaped microreactor was heated from ambient temperature to 800 °C (at heating rate of 10 °C/min) under a flow of H_2_/Ar gas mixture (5/95 vol%, 25 mL/min). Prior to the experiment, the sample was degassed in a nitrogen flow at 100 °C for 1.5 h, and then cooled to RT. The H_2_-TPR profiles were recorded using a thermal conductivity detector (TCD).

### 3.8. FT-IR Spectroscopy Studies of Probe Molecules Sorption

The acidic feature evaluation, both in the quantitative and qualitative manner, was obtained from IR studies of pyridine (Py ≥ 99.8%, Sigma-Aldrich, St. Louis, MO, USA) and carbon monoxide (CO ≥ 99.99, PRAXAIR, Danbury, CT, USA) adsorption experiments, respectively. Prior to the IR experiments the catalysts were evacuated in situ in an IR cell for 1 h at 450 °C, which fall in the temperature range used in the catalytic testing of the materials. The excess of Py was dosed on the catalyst at 170 °C, then the gaseous and physisorbed Py molecules were removed by evacuation at the same temperature. The 1545 cm^−1^ (pyridinium ions, PyH^+^) and the 1450 cm^−1^ (Py coordinatively bonded to the Lewis sites, PyL) bands intensities were applied for estimating the Brønsted and Lewis sites concentration together with their absorption coefficients, i.e., 0.07 cm^2^·μmol^−1^ for the 1545 cm^−1^ band and 0.10 cm^2^·μmol^−1^ for the 1450 cm^−1^ band [82].

The Py thermodesorption experiments aimed at description of the strength of both types of sites were realized by looking at the preservation of the 1545 cm^−1^ band (PyH^+^ ions) upon desorption at 450 °C. The ratio Py_450_/Py_170_ (Py_450_ and Py_170_ are intensities of PyH^+^ ions band upon evacuation at 450 and 170 °C, respectively) indicates for the number of pyridine molecules still neutralizing protonic sites at 450 °C, thus expresses their strength [83]. The nature of the Lewis acid sites was investigated by the low-temperature (−140 °C) sorption of CO in FT-IR experiments [84].

The FT-IR experiments were performed on InvenioX spectrometer (Bruker, Billerica, MA, USA) with the use of custom-made quartz cell. The spectra were collected with use of HgCdTe (MCT) photovoltaic detector. Each spectrum consisted of 100 scans with the resolution of 2 cm^−1^.

### 3.9. Operando DR UV-vis Spectroscopy Studies

The catalysts were subjected to operando DR UV-vis (diffuse reflectance) studies and 2D COS (two-dimensional correlation spectroscopy) analysis. The V zeolites in the form of the self-supported discs were placed in Praying Mantis^®^ coupled with UV-vis spectrometer (Shimadzu UV-2600, Shimadzu Corporation, Kyoto, Japan). The samples were heated at a rate of 6°C/min gradually to 100 °C, 150 °C, 200 °C, 250 °C, 300 °C, and 400 °C in a flow of synthetic air (30 mL/min) and the temperature was maintained for 10 min at each temperature step. Additionally, at 400 °C for 10 min the flow was changed for propane. At least ten spectra were collected at each stage of the process.

The UV-vis spectra collected within the propane transformation over oxidized surface of both V-catalysts were subjected to the 2D COS analysis [85,86,87]. The 2D UV-vis maps consist of much better-resolved peaks and allow for differentiating the subtle differences in the time-dependent intensity variations of the UV-vis bands which are poorly distinguishable in 1D spectra. The positive correlation between the two separate wavelengths is attributed to the simultaneous increase or decrease in intensity of these bands. When the negative correlation occurs, one of the bands changes its intensity at the expense of the other one.

### 3.10. Catalytic Tests

The oxidative dehydrogenation of propane was carried out in a fixed bed gas flow stainless steel reactor in the temperature range 400–500 °C according to the procedure applied earlier [35]. Analysis of substrates and products was performed by online gas chromatography using GC Agilent technologies 7890B (Agilent, Santa Clara, CA, USA) with TCD and FID detectors, equipped with three capillary columns for qualitative and quantitative measurements. The reaction mixture contained 7.1 vol% of propane in synthetic air. The catalysts’ grains of 0.63–1 mm diameter (about 0.5 cm^3^) were used for the catalytic tests, diluted with acid-washed quartz beads of the same diameters (1:1), in order to avoid temperature and concentration gradients. The 0.5 cm^3^ segments of samples (ca. 0.23 g) were mixed with 0.5 cm^3^ (ca. 0.48 g) of quartz grains (grains diameter = 0.63–1 mm), thus obtaining a catalyst layer thickness of 0.89 cm. The microscopically determined diameter of the crystallites was 1.49 × 10^−5^ cm. Under these conditions, the van den Bleek criterion of diluting the catalyst with the inert material was fulfilled [88]:

$$\frac{2.5\;b\;\cdot \;d_{p}}{\left(1-b\right)\cdot \;L_{p}}=1.49\times 10^{-5}<\;5\times 10^{-2},$$

where *b* is the inert bed fraction, *d_p_* is the diameter of catalyst particles, and *L_b_* is the bed length. Analysis of the products and unreacted alkane was started after 1 h of stabilization in the reaction mixture at the given temperature.

The selectivity to the given reaction product, *i*, was calculated from the number of moles of the product *i* divided by the total number of moles of products in the product mixture using the general formula, as follows:

$$S_{i}\left[\%\right]=\frac{x_{i}^{-1}n_{i}}{\sum x_{i}^{-1}n_{i}}\cdot 100$$

while the conversion was calculated as:

$$conv\;\left[\%\right]=\frac{\sum x_{i}^{-1}n_{i}}{\sum x_{i}^{-1}n_{i}+n_{C3H8}\left(output\right)}\cdot 100$$

where $x_{i}$—stoichiometric coefficient of the reaction leading to the product *I*; $n_{i}$—number of moles of the product *I*; $n_{C3H8}\left(output\right)$—number of moles of propane at the output.

For the empty reactor, the conversion of propane was equal to zero in the whole range of temperatures. When the reactor was filled with 1 cm^3^ quartz beads no propane conversion was detected below 500 °C with gas flow of 30 mL/min. At 500 °C the propane conversion was measured to reach 3%.

## 4. Conclusions

Our studies showed that the modification of zeolite via caustic treatment can be effective method of adjusting its lattice oxygen basicity—a key parameter that plays a particularly important role in the ODH process. The significantly developed mesopore surface ensures the attachment of vanadium species to the silanol groups and the formation of the isolated (SiO)_2_(HO)V=O and (SiO)_3_V=O sites or polymeric, highly dispersed forms located in the zeolite micropores. Higher basicity of the lattice oxygen in V-HY_deSi_, as compared to the V-HY, resulting from the presence of the Al-rich shell, aids the activation of the C−H bond activation and higher selectivity to propene. The re-aluminated species also had a positive effect on anchoring the V species to the catalyst surface and affecting the reducibility of the catalyst, while the inferior strength of the protonic sites inhibited the polymerization of propene and the formation of coke compounds. On the basis of the operando DR UV-vis spectroscopic studies, it can be concluded that the bridging V-O-V species anchored to the Al-enriched zeolite surface are highly desirable for the ODH process in low-temperature regime. At the same time, the (SiO)_3_V=O species are ineffective in propane oxidation unless the temperature exceeds 400 °C. In ODH process followed by the in situ FT-IR spectroscopy the formate, acetate, acrylate, carbonates, and water were recognized among the adsorbed species. The stability of alkoxide species was found as significantly lower in V-HY_deSi_, which allows for assigning the catalytic activity to the vanadium sites anchored to the Al-enriched surface of the desilicated zeolite.

## Figures and Tables

**Table 1:** Chemical composition and textural properties derived from N_2_ physisorption. Acidity characteristics is derived from Py and CO adsorption FT-IR studies.

Material	Si/Al ^a^	S_BET_^b^	S_micro_^c^	S_meso_^e^	V_micro_^c^	V_meso_^d^	BAS ^f^	LAS ^f^	LAS_V_	LAS_V_/m^2^	LAS_V_/V_total_	BAS Py_450_/Py_170_^g^
		m^2^·g^−1^	m^2^·g^−1^	m^2^·g^−1^	cm^3^·g^−1^	cm^3^·g^−1^	µmol·g^−1^	µmol·g^−1^	µmol·g^−1^	µmol g^−1^·m^−2^	µmol cm^–3^	[-]
HY	31	883	724	158	0.30	0.22	208	95	-	-	-	0.31
HY_deSi_	18	688	265	423	0.12	0.53	170	180	-	-	-	0.20
V-HY	31	775	645	130	0.23	0.28	145	221	126	0.16	0.14	0.15
V-HY_deSi_	18	319	190	129	0.05	0.35	80	285	105	0.30	0.12	0.08

^a^ Concentration of Al obtained from chemical analysis (ICP). ^b^ Calculated via BET method with the recommendations of Rouquerol et al. [5]. ^c^ Calculated via the t-plot method. ^d^ Volume of primary mesopores (V_p_) and total pore volume (V_t_). ^e^ Calculated as the difference between S_BET_ and S_micro._^f, g^ Data derived from Py adsorption IR studies: ^f^ the concentration of Brønsted (BAS) and Lewis (LAS) acid sites, ^g^ the acid strength of Brønsted acid sites (Py_450_/Py_170_).

## Data Availability

Data are available upon reasonable request from the corresponding authors.
